# Panretinal Optical Coherence Tomography

**DOI:** 10.1109/TMI.2023.3278269

**Published:** 2023-10-27

**Authors:** Shuibin Ni, Thanh-Tin P. Nguyen, Ringo Ng, Mani Woodward, Susan Ostmo, Yali Jia, Michael F. Chiang, David Huang, Alison H. Skalet, J. Peter Campbell, Yifan Jian

**Affiliations:** Department of Biomedical Engineering, Oregon Health & Science University, Portland, Oregon 97239, USA; Casey Eye Institute, Oregon Health & Science University, Portland, Oregon 97239, USA; Casey Eye Institute, Oregon Health & Science University, Portland, Oregon 97239, USA; School of Engineering Science, Simon Fraser University, Burnaby, British Columbia V5A 1S6, Canada; Casey Eye Institute, Oregon Health & Science University, Portland, Oregon 97239, USA; Casey Eye Institute, Oregon Health & Science University, Portland, Oregon 97239, USA; David Huang, and Yifan Jian are with the Department of Biomedical Engineering, Oregon Health & Science University, Portland, Oregon 97239, USA; Casey Eye Institute, Oregon Health & Science University, Portland, Oregon 97239, USA; National Eye Institute and National Library of Medicine, National Institutes of Health, Bethesda, Maryland 20892, USA; David Huang, and Yifan Jian are with the Department of Biomedical Engineering, Oregon Health & Science University, Portland, Oregon 97239, USA; Casey Eye Institute, Oregon Health & Science University, Portland, Oregon 97239, USA; Casey Eye Institute, Oregon Health & Science University, Portland, Oregon 97239, USA; Knight Cancer Institute, Oregon Health & Science University, Portland, Oregon 97239, USA; Department of Radiation Medicine, Oregon Health & Science University, Portland, Oregon 97239, USA; Department of Dermatology, Oregon Health & Science University, Portland, Oregon 97239, USA; Casey Eye Institute, Oregon Health & Science University, Portland, Oregon 97239, USA; Department of Biomedical Engineering, Oregon Health & Science University, Portland, Oregon 97239, USA; Casey Eye Institute, Oregon Health & Science University, Portland, Oregon 97239, USA

**Keywords:** Axial length, handheld OCT, panretinal OCT, retinoblastoma, retinopathy of prematurity

## Abstract

We introduce a new concept of panoramic retinal (panretinal) optical coherence tomography (OCT) imaging system with a 140° field of view (FOV). To achieve this unprecedented FOV, a contact imaging approach was used which enabled faster, more efficient, and quantitative retinal imaging with measurement of axial eye length. The utilization of the handheld panretinal OCT imaging system could allow earlier recognition of peripheral retinal disease and prevent permanent vision loss. In addition, adequate visualization of the peripheral retina has a great potential for better understanding disease mechanisms regarding the periphery. To the best of our knowledge, the panretinal OCT imaging system presented in this manuscript has the widest FOV among all the retina OCT imaging systems and offers significant values in both clinical ophthalmology and basic vision science.

## I. Introduction

Many vitreoretinal disorders can manifest with morphological abnormalities in both the posterior pole and the peripheral retinal capillary network at an early stage [1]–[6]. In adult retina, most tractional and rhegmatogenous retinal detachment are due to peripheral pathology, such as extraretinal neovascularization, membranes, and retinal breaks. In addition, much of the pathology in common retinal vascular diseases such as diabetic retinopathy involve the peripheral retina. Infants with diseases that affect the peripheral retina, such as retinopathy of prematurity (ROP), Coats disease, retinoblastoma, etc., are often not noticed until their vision in one or both eyes is severely impaired. Imaging technology with adequate visualization of both central and peripheral regions of the retina is desirable for early diagnosis and treatment of these eye diseases, may prevent visual impairment, and could even improve mortality rates such as in patients with retinoblastoma. However, capturing images of peripheral retina has been challenging, especially in pediatric populations. In addition to visualization of retinal periphery, ocular biometry (measurement of axial eye length) is required for quantitative measurement of anatomic features on the retina and may aid the understanding of retinal disease and evaluating eye health.

There are very limited commercially available retinal imaging devices that can visualize peripheral retina. Scanning laser ophthalmoscopes (SLO) such as Optos ultra-widefield retinal imaging and Heidelberg SPECTRALIS with ultra-widefield module have a field of view (FOV) up to 200° (measured from the center of the eyeball) and are frequently used in ophthalmic clinics. These imaging devices are in desktop format, bulky and require patient cooperation including maintaining a steady position of gaze. It is possible to image infants with these desktop systems in so-called “flying baby position” in which they are held by the photographer. However, this requires significant operator skills and experience, and is not widely available. Handheld fundus cameras (RetCam Envision, Natus Medical Inc., CA) are increasingly being used in managing pediatric retinal diseases due to their portability. Compared to the scanning laser ophthalmoscopes, fundus cameras have inherently low contrast and resolution due to the lack of confocal pinhole to reject scattered light. Another limitation of portable fundus cameras is their smaller FOV, limited by the size and illumination scheme.

Optical coherence tomography (OCT) is a non-invasive imaging technology used to acquire detailed information on ocular structures by the interferometric detection of the back reflected light [7]. OCT has revolutionized ophthalmic imaging and transformed clinical practice in ophthalmology. However, most of the OCT retinal imaging systems including both commercially available units and research prototypes do not provide imaging FOV over 100° [8]–[13]. In conventional implementation of a retinal OCT system, it is possible to adjust the combination of the focal length and diameter of the lens in the eyepiece to obtain a relatively large FOV [8], [14]. However, the working distance limits this strategy when the FOV is over 100°. Alternatively multiple images can be stitched together. This method relies on patient cooperation to adjust gaze toward various target points to capture the peripheral fields. This issue, together with the need for a particularly skilled operator, and extended acquisition time make the stitched image solution impractical. In general, desktop OCT retinal imaging systems require patients to fixate and maintain their postures steady, which significantly limits their use in imaging infants and small children. To address this issue, several groups have reported on handheld OCT prototypes for imaging patients in the supine position [9]–[13], [15]–[20]; however, most of these portable prototypes cannot capture the posterior pole and peripheral retina in a single image, which significantly limited their clinical applications.

Beyond the challenges in acquiring useable widefield retinal images, another limitation for the above-mentioned retinal imaging devices is that these devices cannot accurately measure the anatomical features on the retina, due to the lack of eye axial length (AL) measurement. AL measurement allows for reliable quantitative analysis of ocular structures, which is important for accurate diagnosis and monitoring of eye diseases. A-scan ultrasound biometry has traditionally been the most used method for AL measurement but comes with the limitation of poor image resolution [21], [22]. OCT imaging technology can provide much higher image resolution and has been used in ocular biometry measurement including axial eye length [23], [24]. However, conventional OCT is not suitable for AL measurement due to the limited imaging depth and displacement error of the scanning pivot point during image acquisition.

The inability to capture the peripheral retina efficiently and reliably and the absence of quantitative evaluation of changes in the peripheral retina have limited the thorough investigation and diagnosis of peripheral retinal diseases. In this manuscript, we introduce a contact handheld swept-source OCT (SS-OCT) system with an unprecedented single shot 140° FOV, and demonstrate its use in a variety of retinal diseases. Imaging with a contact-based approach allows maximal control over eye position, eliminates the need to manually adjust the scanner’s working distance, and is familiar to photographers and clinicians who manage pediatric retinal diseases and utilize fundus photography. With the new contact imaging probe design, we were able to significantly reduce the time needed for alignment, improve image quality, and achieve higher success rate than our previous handheld OCT imaging systems [11], [13], [25]. Importantly, the utilization of a contact approach also allows quantitative analyses of retinal pathology to further our understanding of morphologic and functional aspects of various retinal diseases. To the best of our knowledge, this is currently the widest FOV achieved among all retinal OCT research prototypes and commercial systems in desktop and portable format.

## II. Methods

### A. System Setup

The swept-source laser in the system had an A-scan sweep rate of 400 kHz, 50% duty cycle, a center wavelength of 1060 nm, and ~70 nm bandwidth (6 dB), which corresponded to an axial resolution of 7.06 μm in air. Each A-scan spectrum was sampled by 2048 points corresponding to a peak sampling frequency of 1720 MHz and an imaging depth of 6 mm in air. The beam size on the pupil plane was 0.36 mm, resulting in a theoretical beam diameter of 45.3 μm (1/e^2^) on the retina plane when imaging an infant with an AL of 17 mm. The output power from the handheld probe was 1.68 mW, which is well below the ANSI Z136.1-2013 standards for 1060 nm light [26].

**Fig. 1:**
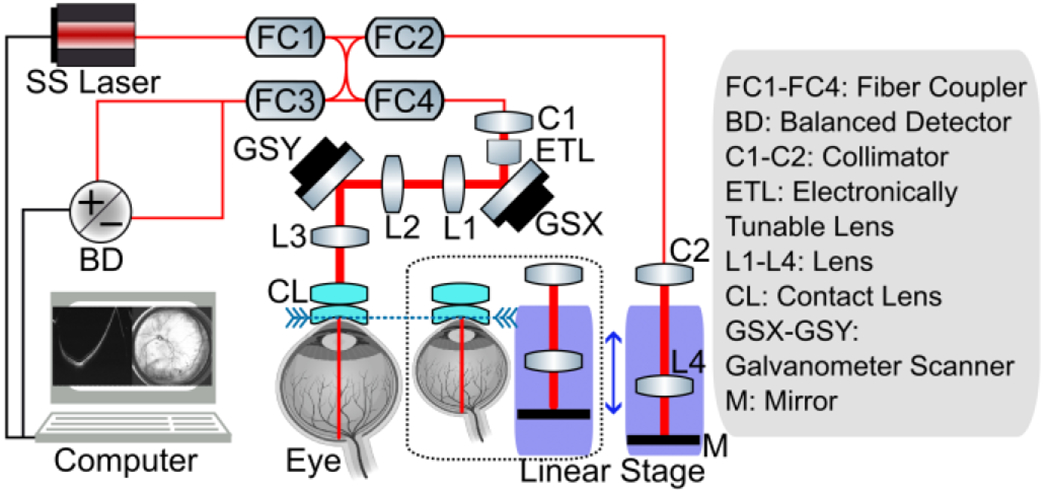
Schematic of contact handheld panretinal SS-OCT system. Swept-source laser (SVM10F-0210, Thorlabs, Inc., USA); Split ratio of fiber coupler: FC1 (10/90), FC2 (50/50), FC3 (50/50), FC4 (20/80); Galvanometer scanner (Pangolin Laser System, Inc. USA); Balanced detector (PDB482C-AC, Thorlabs, Inc., USA); Linear stage (X-LHM100A-E03, Zaber Technologies, Inc., Canada).

A schematic diagram of the contact handheld panretinal SS-OCT system is shown in Fig. 1. A software-controlled linear stage allowing high precision positioning was integrated into the system to adjust the length of the reference arm. The motorized translational stage with built-in encoders provided position information, which was recorded along with the OCT images. The stage had an adjustable length range of 100 mm, allowing optical path length matching on both the cornea and the retina planes in patients of different eye sizes. An electronically focus tunable lens (EL-3-10, Optotune, Switzerland), which had correction focal length range from −77 mm to 77 mm, was placed after the collimator to adjust the focal length dynamically.

### B. Portable Probe Setup

**Fig. 2:**
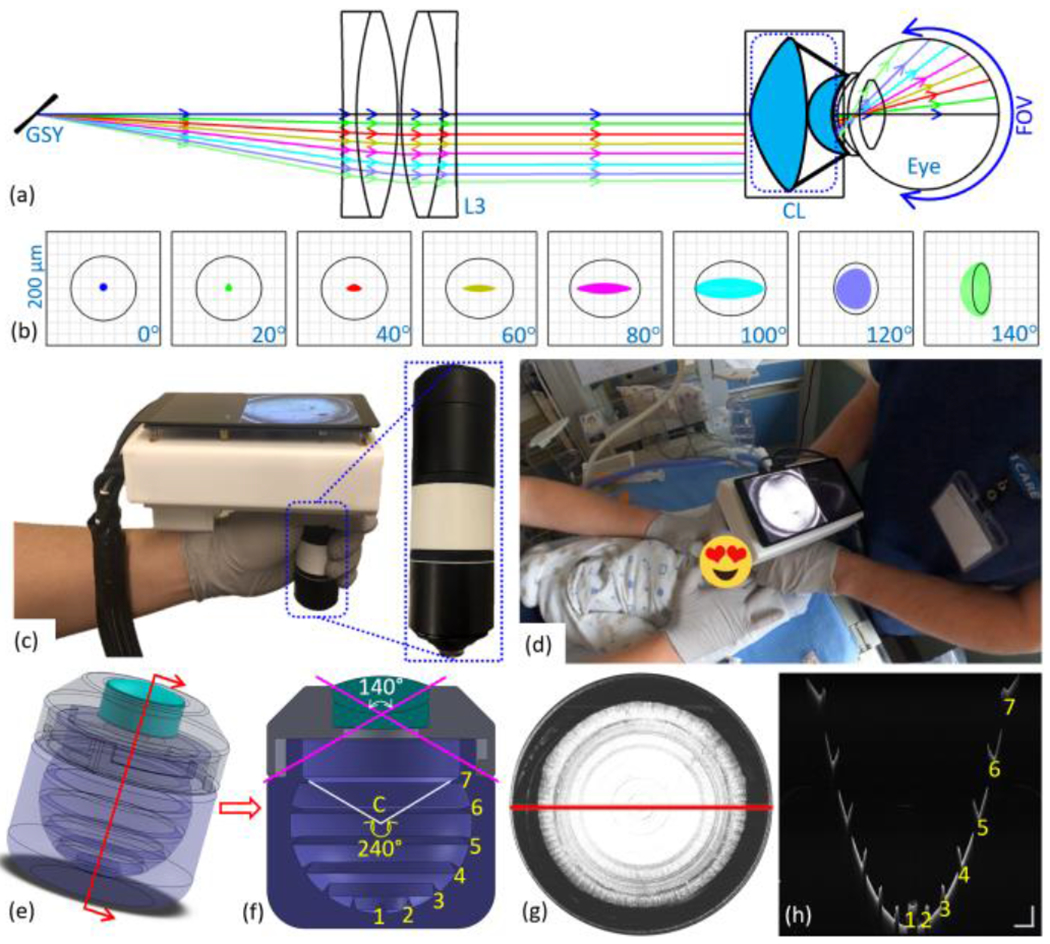
(a) 3D layout of telescope eyepiece design after the slow axis of the galvanometer scanner (GSY). (b) Spot diagrams of FOV from 0° to 140°. Black circle is the Airy disk. The complex double aspherical ocular lens induced some irregular beam profile on the retina. (c) Photograph of the fully assembled portable probe. (d) Photograph of contact handheld SS-OCT being used to image premature infants in the OHSU neonatal intensive care unit. (e) SolidWorks rendering of phantom eye model for FOV calibration (radius = 12 mm). (f) Cross-sectional view of the phantom eye. Seven circular rings extruded from the surface were marked. The interval between the rings was 40° measured from the center of the phantom eye (point “C”). (g) *En face* OCT image from the phantom eye. (h) Selected cross-sectional scan corresponding to the location of red line in (g). Scale bars in (h) are 1 mm.

The transverse resolution of the OCT systems is determined by the numerical aperture (NA), and the optical aberrations in the imaging system and the ocular media. A retinal imaging system free of optical aberrations is diffraction limited, which is predominantly determined by the beam size on the pupil plane and the refraction power of the eye. Ocular aberrations can vary significantly from person to person and can only be corrected by adaptive optics. However, in a low NA retinal imaging system, the ocular aberrations do not significantly impair the transverse resolution. While it is possible to design a diffraction-limited OCT imaging system with low NA over a moderate FOV, this task becomes increasingly difficult as the FOV expands. To meet the demand for ultrawide FOV, we first separated and placed the two scanners on the pupil conjugate planes with a set of relay telescope (L1 and L2 in Fig. 1, 4×AC254-035-B, Thorlabs Inc., USA). This ensured that the scanning beam pivot points of the two axes were overlapping, hence reducing vignetting artifact. The telescope eyepiece was constructed with a pair of achromatic doublet lenses (L3 in Fig. 1, 2×AC254-100-B, Thorlabs Inc., USA) and a contact ocular lens [CL in Fig. 1 and Fig. 2(a), Quad Pediatric, Volk Optical Inc., USA]. The Quad Pediatric lens was composed of an advanced double aspheric lens and a meniscus lens [Fig. 2(a)]. The curvature of the meniscus lens was specifically designed for pediatric patients with small eyes, which maintained stability while ensuring the patients’ comfort. The Quad Pediatric lens was commonly used in the application of indirect ophthalmoscopy with white light illumination. Therefore, the commercially available version has an anti-reflection (AR) coating for the visible wavelength range, which would greatly reduce the optical power when used in OCT imaging at 1060 nm. To improve the transmission in the infrared wavelength range, AR coating with reflectivity < 0.5% from 950 nm to 1150 nm was applied on all the optical surfaces of the Quad Pediatric lens.

The novel optical design was developed and optimized in OpticStudio (Zemax, LLC, USA). The spot diagrams were used to demonstrate and quantify the system’s performance. Several angular positions from 0° to 140° were chosen to investigate the influence of different FOVs on the performance of the system [Fig. 2(b)]. The optimized Zemax simulation model was imported into CAD software (SolidWorks, Dassault Systemes, France) for developing the mechanical design. The precise control over the distance between the lenses was delivered by the 3D printing [white parts in Fig. 2(c)], which ensured that the system was always in optimum performance when the telescope eyepiece was removed for routine maintenance without the need of realignment.

### C. Volumetric Imaging Field of View

**Fig. 3:**
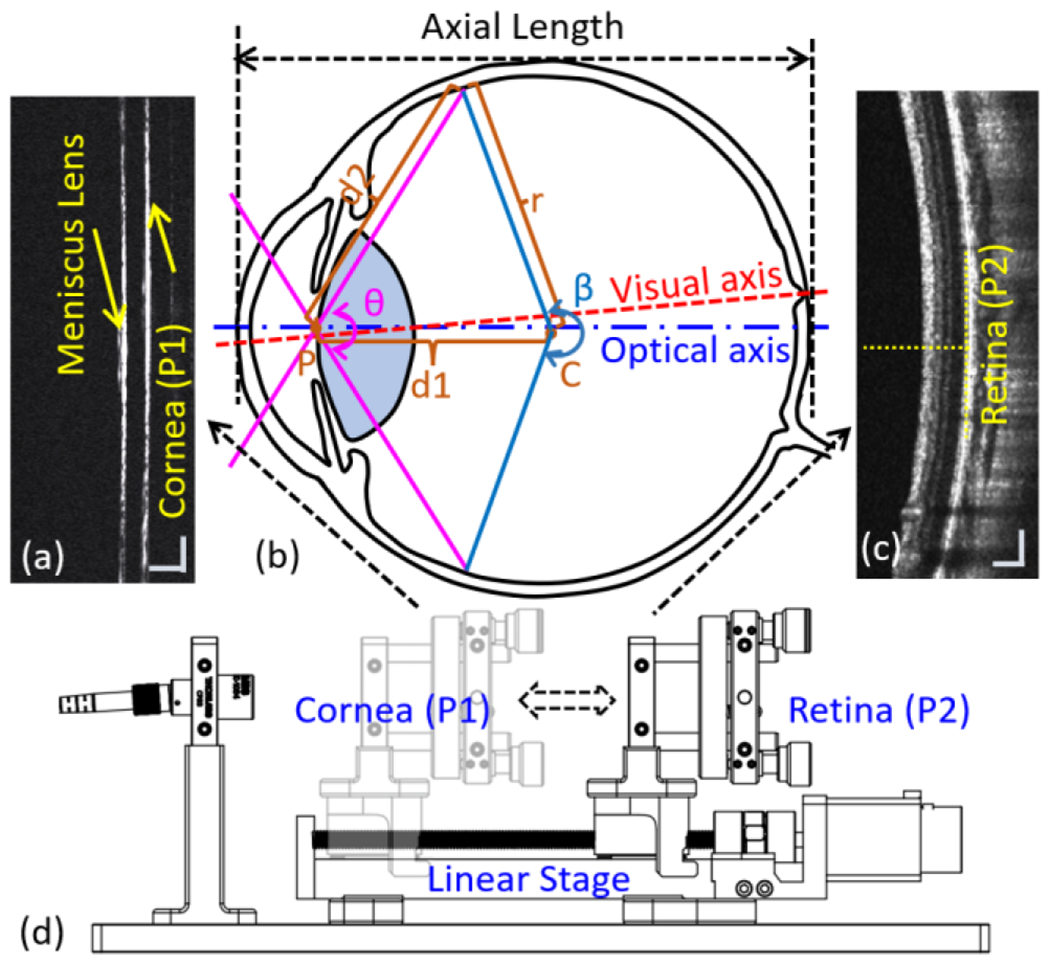
(a) Cross-sectional scan of the meniscus lens and corneal surface. (b). Schematic diagram of the human eye. P: scanning pivot point. θ: scanning angle. β: FOV measured from the center of the eyeball. C: center of the eye. r: radius of the eye. (c) Cross-sectional scan of the retina. (d) Schematic of the reference arm indicating with the linear stage, stopped at the corneal position (P1) and retinal position (P2). Scale bars in (a) and (c) are 200 μm.

The placement of the scanning pivot point [point “P” in Fig. 3(b)] is of particular significance in the application of the panretinal OCT imaging system affecting the scanning FOV in all three dimensions. On the axial dimension, the light ray path difference between the central and the most peripheral ray [(*r* + *d*1 − *d*2) in Fig. 3(b)] determines the required imaging depth to fully accommodate the retina area being imaged. In this study, the eyeball was assumed to be an ideal sphere to simplify the model. At the same scanning angle [angle “θ” in Fig. 3(b)], as the scanning pivot point moved posteriorly towards the retina, the curvature of the retina rendered in the cross-sectional scan would reduce, hence the required imaging range to fit the entire retina being scanned in one cross-section scan without aliasing artifact would gradually shorten. In general, cross-sectional scans have a flatter curvature when the scanning pivot point is placed towards the center of the eye [point “C” in Fig. 3(b)]. In addition, the flatter retinal curvature would allow moving the entire cross-sectional scan towards the low frequency part where the signal roll-off is at minimum, thus having better signal-to-noise ratio (SNR). However, in the panretinal OCT imaging, if the scanning pivot point is placed close to the center of the eye as in conventional OCT imaging, the OCT scanning beam entering the eye at large scanning angles will be blocked by the iris, reducing the transverse FOV even when the pupil is fully dilated. With contact approach we could easily place the scanning pivot point on the iris plane without manual adjustment of the working distance and achieve optimum transverse FOV. Commercial fundus camera (RetCam Envision, Natus Medical Inc., CA) and scanning laser ophthalmoscope (Optos Inc., UK) measure the FOV from the center of the eyeball, whereas in our panretinal OCT scanner, we define our FOV as the scanning beam pivoting angle on the iris plane.

### D. Scanning Protocol and Data Visualization

OCT images were acquired, processed, and displayed in real-time by our custom software OCTViewer, which was accelerated with a graphics processing unit (GPU) [27]–[29]. OCTViewer offers two scanning patterns, a high-speed alignment mode and a high-resolution data acquisition mode. The operator could toggle between these two modes as needed. The high-speed alignment mode operated at a 10 Hz volume rate with 400 A-scans per B-scan and 100 B-scans per volume. This scan rate provided real-time feedback, allowing us to locate the area of interest. Compared with the non-contact approach [25], the contact approach significantly reduced the alignment time due to the pre-setting of the scanning pivot point without needing to adjust the working distance. Once the area of interest was found, autofocus was performed within 1 s based on the brightness of the *en face* images using a hill-climbing algorithm [30]. When sufficient image brightness and contrast is achieved and patient motion is at minimum, the operator could switch to the high-resolution data acquisition mode to save multiple raw OCT volumes for redundancy and quality control. In this mode, the scanning protocol consisted of 800 A-scans per B-scan and 780 B-scans per volume. Each volume acquisition time was 1.56 s. The short acquisition time could ensure the successful acquisition of images with minimum motion artifacts.

### E. Axial Length Measurement

Pediatric eye AL growth is essential for monitoring the progression of childhood eye pathologies. The relationship between AL growth in infants and the severity of ROP has previously been discussed [31]. Moreover, AL growth is also a crucial parameter for monitoring myopia progression [32]. To assess AL in the myopia progression and monitor the eye growth of premature infants, accurate quantification of the morphological characterization is critical. Long-range SS-OCT with imaging depth covers from the anterior chamber to the posterior pole have been demonstrated for optical biometry [33]. However, due to the digitizer speed and bandwidth limitations, long-range OCT systems usually have a low A-scan sweeping speed, which is not ideal for handheld panretinal imaging applications. When the imaging depth could only cover part of the eye, a non-contact OCT imaging approach cannot accurately determine the AL due to the lack of a fixed reference plane. In the contact handheld OCT system, the position of the corneal surface and the scanning pivot point were fixed at each imaging session, which could ensure the repeatability of measurements.

Unlike ultrasound, OCT measures the optical path length (OPL) rather than the acoustic path length to convert the eye’s geometry length (GL). When converting GL from OPL, the mean refractive index (similar to the application of mean velocity in ultrasound) has to be applied [33]. The optical parameters of the Gullstrand eye model of newborn infants were listed in Table I [34], [35]. The average refractive index was calculated based on the formula

$$n_{\mathrm{avg}}=\frac{1}{d}\overset{5}{\underset{i=1}{\sum }}n_{i}\times d_{i}$$

where *n_avg_* is the average refractive index, *d* is the axial eye length, *n_i_* is a refractive index at different ocular segments, and *d_i_* is the thickness of different ocular segment. The result is *n_avg_* = 1.357.

The OPL change is the result of the GL change in the eye. Therefore, the average refractive index of the eye *n_avg_* is also given by the OPL divided by the GL, which is

$$n_{\mathrm{avg}}=\frac{\mathrm{OPL}}{\mathrm{GL}}$$

The position of the meniscus lens was fixed and pre-recorded [Fig. 3(a)]. At the beginning of each imaging session, the reference arm position and the focus of the imaging beam were first adjusted on the corneal surface. OCT images and the reference arm position for the corneal surface were recorded. Then the reference arm was rapidly increased to match the retina position, where OCT data and reference arm position, where OCT data of the retina along with the reference arm position were saved as well. After the imaging session, the OPL was determined according to the positional difference between the corneal apex and fovea along the visual axis [Fig. 3(b)].

### F. Study Subjects

The 140° FOV contact handheld SS-OCT was used to image infants in the operating room and neonatal intensive care unit (NICU). These subjects were recruited from the Casey Eye Institute at Oregon Health & Science University (OHSU) between August 2021 and May 2022. Written informed consent for imaging from the infant’s parents or guardians was obtained prior to initiating the study. The protocol was approved by the Institute Review Board/Ethics Committee of OHSU and adheres to all tenets of the Declaration of Helsinki.

## III. Results

Sixty-seven infant subjects (Table II) were recruited in this study, and 246 imaging sessions were performed with 99.6% of infants imaged successfully. Imaging was deferred in the setting of clinical factors that would make imaging too time consuming and stressful for the infants, such as swollen eyelids due to continuous positive airway pressure (CPAP).

Each imaging session, including an alignment process and image acquisition, was optimized to complete within 2 minutes to reduce the discomfort of the patients. Before the imaging session, the subject was dilated with cyclopentolate hydrochloride and phenylephrine hydrochloride. Lubricant eye gel (Systane, Alcon, Switzerland) was applied to the cornea to prevent corneal abrasion to facilitate coupling of the meniscus lens with the cornea. An eyelid speculum was placed to keep the eye open and ensure sufficient contact between the ocular surface and the meniscus lens, which could greatly speed up the imaging procedure. The meniscus lens was sterilized before each imaging session. During the imaging session, the operator gently rested the hand on the forehead of the baby to precisely control the pressure on the eyeball while imaging. A complete imaging session was recorded and shown in Visualization 1. When imaging uncooperative patients such as young children and infants with handheld retina scanner, motion artifacts can be severe and have significant impact on the image quality. Through the combination of high-speed laser, fast scanning pattern, real-time image feedback as well as contact lens design, we were able to minimize the influence of motion artifacts in our handheld panretinal OCT system.

### A. Retinoblastoma

Retinoblastoma is a rare and aggressive intraocular cancer in young children. Mortality from retinoblastoma is less than 5% in high-income countries but about 70% in low- and middle-income countries [36]–[39]. There are about 9000 new confirmed cases each year worldwide, and most children do not survive [37], [40]. Retinoblastoma can occur anywhere on the retina and patients may develop additional tumors over time, with a later predilection for peripheral retina [41]. Therefore, it is crucial to evaluate the entire retina and document with imaging as large a region as possible [41]. Delayed detection of tumors in retinoblastoma can compromise patient vision and possibly survival. Currently, magnetic resonance imaging, ocular ultrasound, fundus camera and indirect ophthalmic examination are the most common imaging technologies used to manage retinoblastoma [42]–[44]. These imaging tools are not very efficient due to limitations such as lengthy imaging time, resolution, and FOV. The application of handheld OCT has recently proven valuable in detecting small invisible tumors in retinoblastoma that were not detectable by indirect ophthalmoscopy [45]–[47]. It can also be used to detect subclinical tumor recurrence. However, current commercial handheld OCT devices do not provide FOV sufficient for optimal management of retinoblastoma as many tumors lie in areas that cannot be captured.

**Fig. 4:**
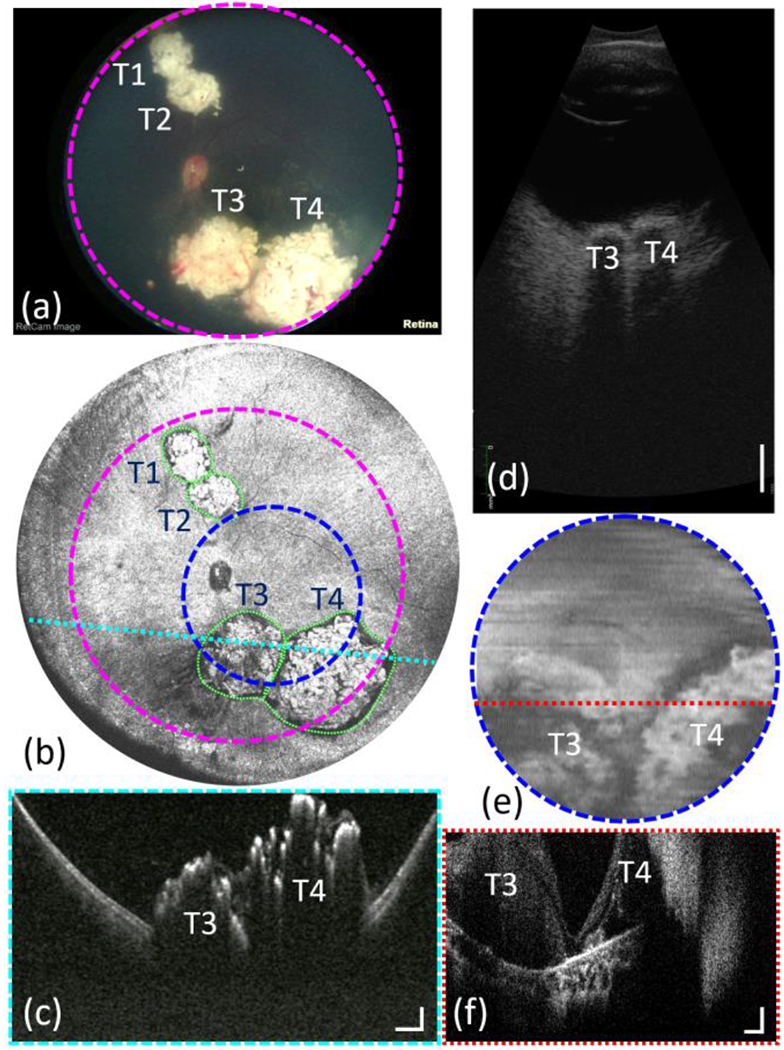
(a) RetCam color fundus image from a patient with multifocal retinoblastoma (tumors labeled as T1-T4). (b) *En face* OCT image obtained by our contact handheld SS-OCT system. (c) Selected B-scan image corresponding to the location of dashed cyan line. Scale bars are 1 mm (horizontally) and 500 μm (vertically). (d) Selected ultrasound B-scan image. Scale bar is 5 mm. (e) *En face* OCT image taken by Leica handheld OCT (Envisu C2300, Leica Microsystems, Germany). The imaging area was marked by dashed blue circle in (b). (f) Selected B-scan image corresponding to the location of dashed red line. Scale bars are 500 μm (horizontally) and 200 μm (vertically).

To demonstrate the panoramic imaging capability of our handheld OCT system, an 18-month-old patient with multifocal retinoblastoma was imaged under general anesthesia during routine follow-up care. RetCam color fundus image, representative ocular ultrasound B-scan (Eye Cubed 13 V-4, Ellex, Australia), and study OCT images are shown in Fig. 4. The contact handheld panretinal OCT acquired quantitative three-dimensional images over the retina with significantly wider FOV compared to the commercially available handheld retinal imaging systems, such as Leica Envisu C-Class OCT and Natus RetCam. With Leica HH OCT, small portions of the tumors T3 and T4 were visible on the *en face* image and B-scan, due to the limited FOV and axial imaging range.

For this eye, the AL measured by the ocular ultrasound B-scan was 21.5 mm and by the contact handheld OCT system was 22.14 mm. The ocular ultrasound B-scan that was acquired with the 10 MHz posterior mode had an axial resolution of 50 μm and lateral resolution of 100 μm. The axial eye length measured on the ultrasound B-scan was from the anterior corneal vertex to the internal limiting membrane (ILM); whereas, with the OCT, the OPL was measured up to the retinal pigment epithelium (RPE) [34]. In addition, due to the lack of volumetric imaging capability, it is difficult to identify exactly where the ultrasound B-scan was taken on the retina, and the fovea was not present on the ultrasound B-scan, which also contribute to this discrepancy. In this eye, four retinoblastoma tumors were labeled as T1-T4, as shown in Fig. 4(b). The raw OCT volume data was processed and rendered volumetrically. Then the corresponding sizes of the tumor were fitted according to the retinal curvature. The dimensions of the tumors were calculated and listed in Table III.

In retinoblastoma care, documentation of tumor size and location as well as ongoing monitoring of tumor size and morphology are of critical importance in evaluating response to treatment and identifying tumor recurrence. Quantitative volumetric measurement using the contact handheld panretinal OCT system could significantly improve reliability of clinical assessment for tumor control in retinoblastoma patients and lead to improved outcomes.

### B. Retinopathy of Prematurity

**Fig. 5:**
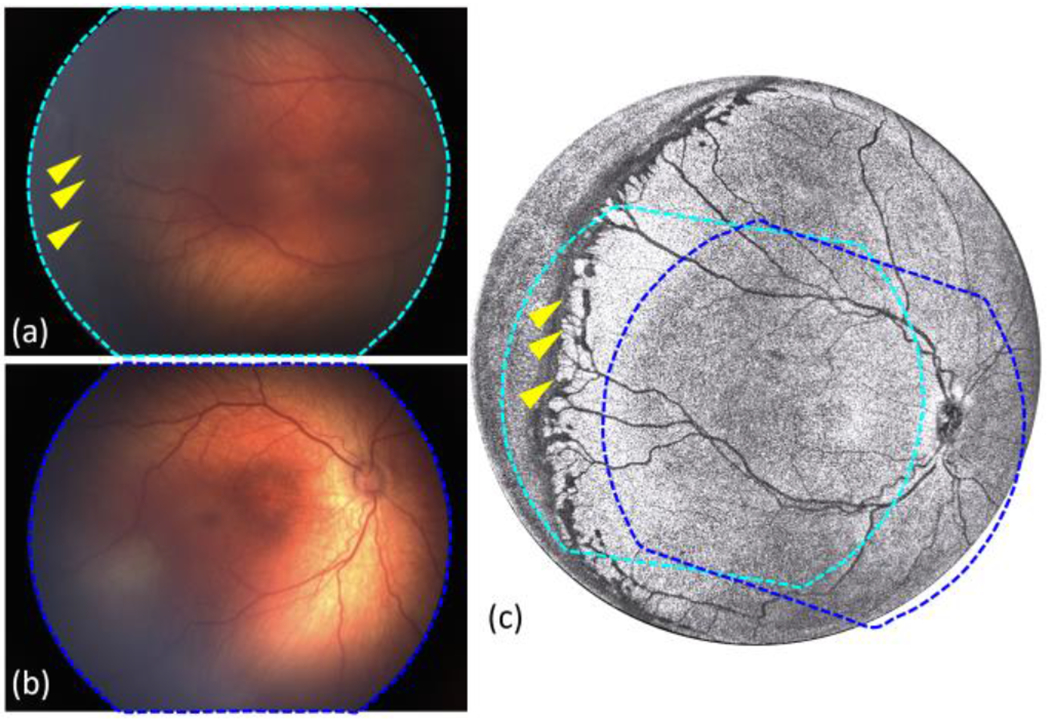
(a)-(b) RetCam color fundus images from an infant with ROP stage 3 at different locations. (c) *En face* OCT image from the same infant acquired by contact handheld panretinal SS-OCT imaging system. Yellow arrows in (a) and (c) point to the area of extraretinal neovascular proliferation.

Stage 3 ROP is associated with significant vascular abnormalities, and delayed diagnosis might lead to blindness [48]. Early detection with an ultra-widefield imaging tool followed by prompt treatment could prevent vision loss. A comparison between RetCam color fundus images and *en face* OCT image from the same premature infant was shown in Fig. 5. The dashed cyan and blue boxes in Fig. 5(c) correspond to Figs 5(a) and 5(b), respectively. The *en face* OCT image covered the retina area extended from the posterior pole to the retinal periphery, which was larger than the single RetCam fundus image, specified to have a 130° FOV. In addition, the image contrast at the area of extraretinal neovascular proliferation (yellow arrows) in the *en face* OCT image is much better than that in the RetCam fundus image.

**Fig. 6:**
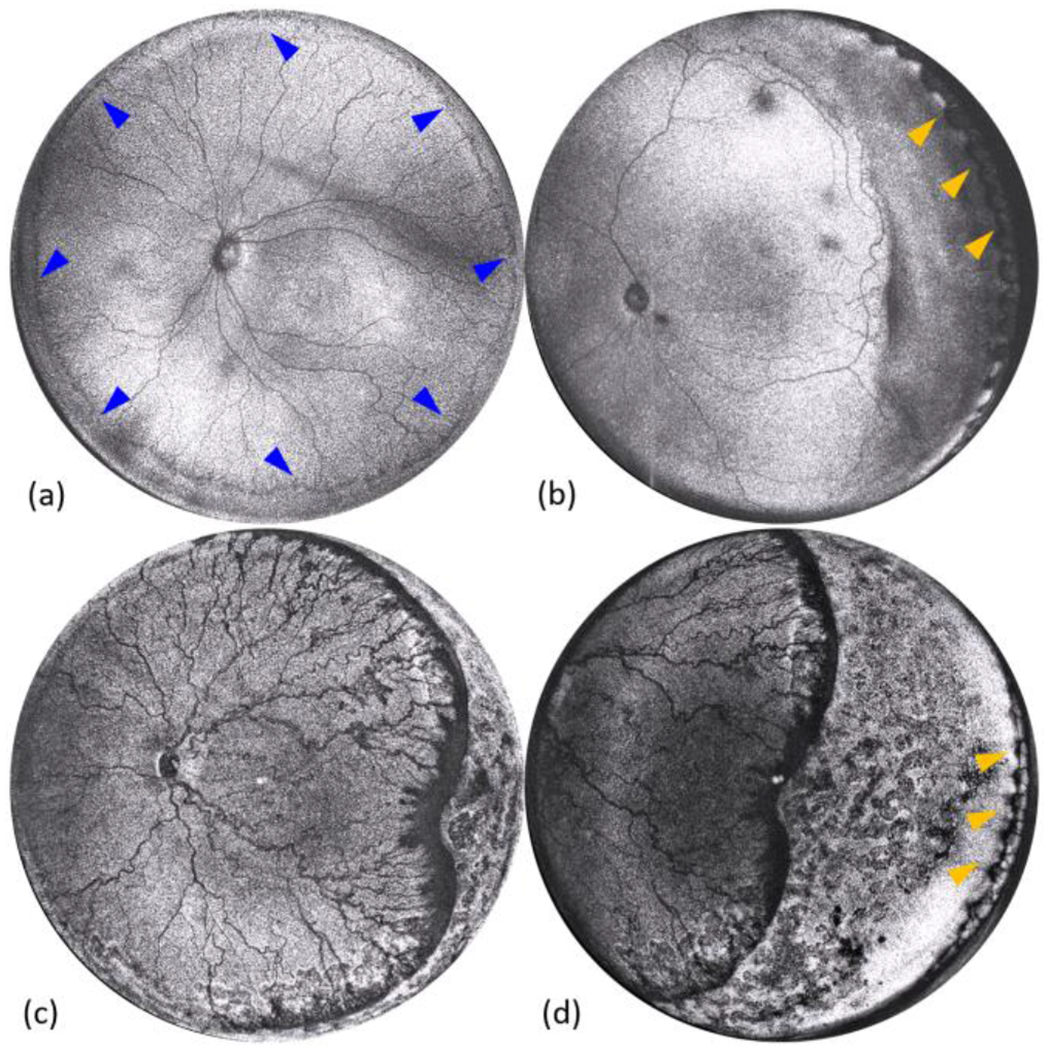
(a) *En face* OCT image from an infant with ROP stage 1. Blue arrows point to the demarcation line with the retinal vascular-avascular junction. (b) *En face* OCT image from an infant with ROP stage 2. The gray streak visible in the image is a motion artifact that occurred during image acquisition. (c)-(d) *En face* OCT images from the infant with ROP stage 3 at different locations after laser treatment. Orange arrows in (b) and (d) point to the area of ora serrata.

*En face* OCT images from three infants with ROP at different stages were presented in Fig. 6. Demarcation line separating the vascular and avascular retina [blue arrows in Fig. 6(a)] was distributed all around zone I. To the best of our knowledge, this was the first time that entire the 360-degree demarcation line was visualized in a single OCT image. Some groups have reported that they could use a montage to take OCT to the peripheral retina. In addition, we were also able to identify the ora serrata on the nasal side by rotating the probe, which was considered more difficult to observe than on the temporal side, as pointed by the orange arrows in Figs 6(b) and 6(d). *En face* OCT images in Figs 6(c) and 6(d) were from the same infant but located in different areas. The utilization of handheld panretinal OCT is beneficial for clinicians in identifying and documenting disorders throughout the retina.

Six subjects with different severities of ROP were selected as representatives to calculate the AL according to the method described in the previous section. The result is listed in Table IV. One of the premature infants (Subject 6) was followed for two months to observe and record the development of the eye.

### C. Coats Disease

**Fig. 7:**
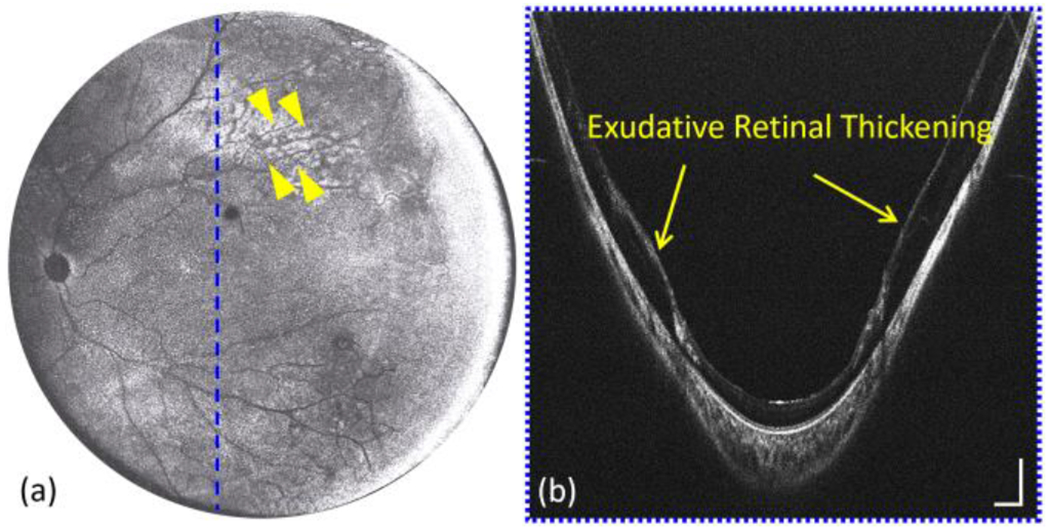
(a) *En face* OCT image from a patient with Coats disease (yellow arrows). (b) Selected cross-sectional scan with the regions of extramacular exudation and retinal thickening corresponding to the location of dashed blue line in (a). Scale bars in (b) are 1 mm (horizontally) and 500 μm (vertically).

Figure 7 demonstrates a representative example of Coats disease acquired by contact handheld panretinal OCT. Coats disease is an idiopathic retinal vascular disease with retinal telangiectasia and can be complicated by several vision-threatening conditions, such as retinal detachment, glaucoma, cataract, etc. [49]. Note that retinal telangiectasia in Coats disease is often associated with exudation, which can lead to exudative retinal thickening as shown in Fig. 7(b), and at times detachment. The utilization of panretinal OCT imaging in the diagnosis of Coats disease allows greater exploration of the retinal periphery, which facilitates identifying the presence of retinal pathology.

### D. Retinal Detachment

**Fig. 8:**
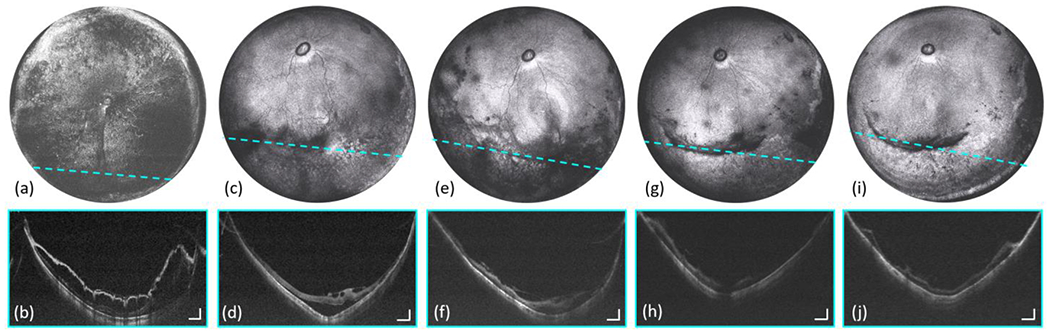
Morphological changes in the retina of the patient with retinal detachment with different therapeutic interventions. The images on the first row are *en face* OCT images. The images on the second row are selected cross-sectional scans. The dashed cyan lines indicate location of the corresponding cross-sectional scans. (a)-(b) One week after panretinal photocoagulation laser treatment. (c)-(d) Three weeks after panretinal photocoagulation laser treatment. (e)-(f) Two weeks after PPV surgery. (g)-(h) Six weeks after PPV surgery. (i)-(j) Seven weeks after PPV surgery. The PPV surgery was performed six weeks after the panretinal photocoagulation laser treatment. Scale bars are 1 mm (horizontally) and 500 μm (vertically).

Retinal detachment is a sight-threatening condition in which the neurosensory retina becomes separated from the retinal pigment epithelium. Prompt identification of the cause of retinal detachment is required to guide treatment [50]. OCT can be used to capture the morphology of retinal elevation, which helps to distinguish between types of retinal detachment and identify underlying breaks or tractional membranes. Figure 8 shows an eye with an exudative retinal detachment [Figs. 8(a)–8(b)], which improved after topical corticosteroid therapy and additional panretinal photocoagulation [Figs. 8(c)–8(d)]. Subsequent to treatment, the patient went on to develop retinal traction, for which a pars plana vitrectomy (PPV) was performed. Figures 8(e)–8(j) demonstrate the utility of OCT to evaluate and monitor retinal repair post-operatively.

## IV. Discussion

We have developed a 400 kHz handheld SS-OCT system with an unprecedented 140° FOV using a contact approach, and demonstrated its significant value in diagnosis and management of disease involving peripheral retina. Our imaging system leverages a state-of-the-art MEMS-VCSEL laser, parallel computing with GPU, and novel optics design to achieve imaging of the entire retina beyond the equator, reaching the ora serrata. The panretinal FOV and high-speed imaging technique has great potential to improve the diagnosis of retinal diseases in adults and infants. This approach is particularly promising for pediatric applications, such as assessment of ROP, and adult patients under sedation. Because premature infants cannot cooperate with the photographer for the imaging adjustment, the contact approach is conducive to minimizing the eye motion artifacts and eliminating shadow artifacts caused by the patient’s eyelashes. The first significant achievement in our prototype is the unprecedented 140° FOV (pivoting angle on the iris plane), which can capture the posterior pole and far peripheral retina in a single shot. In addition to the optical design, UWF imaging systems are limited by the imaging speed. To satisfy the Nyquist sampling theorem, 1) wider FOV demands more sampling points, 2) higher optical resolution entails higher sampling density, both of which require higher imaging speed. In practice, even with ultra-high speed swept source lasers, the lateral resolution is intentionally kept low (in the orders of 50 μm, which is not suitable for imaging detailed vasculature networks with OCT angiography). We have carefully considered the design criteria and trade-offs between the FOV, resolution, and image speed in our panretinal OCT system with priority on the FOV and imaging speed, while sacrificing the transverse resolution. Our novel optical design enables panoramic view of the retina with adequate transverse resolution to visualize the peripheral retinal disease while maintains high imaging speed to complete imaging session quickly, which is critical in minimizing patient discomfort, motion artifacts, and significantly improving success rate and image quality.

The adequate visualization of the periphery has excellent utility in ocular disease surveillance, especially in clinically silent pathologies originating from a peripheral area and are generally not visualized with standard OCT imaging. Some studies regarding disease mechanisms and proof of concept were limited by available imaging technology. For example, early research in ROP utilizing time-domain OCT was often limited to evaluation the macula [51]. With the ability to sufficiently visualize the pathologies in the peripheral retina, we have started to conduct more sophisticated analyses to improve understanding regarding the role that peripheral pathology can play in retinal diseases, for instance, the previously unknown relationship between the peripheral fibrovascular ridge thickness and ROP stage classification [52], [53]. The effect of therapeutic interventions for prevention of severe ROP may also be explored. It is also possible to use contact approach panretinal OCT to image adults for various ocular diseases that involve peripheral retina, including diabetic retinopathy (DR), neovascular and non-neovascular age-related macular degeneration (AMD), vascular occlusion, and retinal detachment [54]–[56]. The slight discomfort of the contact approach could be justified especially in the case of retinal detachment, in which identifying the location and the exact cause of the detachment is critical.

In addition to the ability of comprehensive retinal screening, our handheld OCT with contact lens design enables measurement of axial eye length, a critical parameter required for quantitative evaluation of biomarkers on retina. The contact lens in our OCT probe is a meniscus lens that matches the corneal curvature, a similar concept compared to the immersion method used in ocular ultrasound. By using lubricant eye gel that couples the cornea and the contact lens, and applying very light pressure, we do not expect significant compression exerted by the contact imaging probe. However, it is possible that curvature mismatch and differences in eye sizes may impact image quality for various reasons. Unfortunately, we did not have other meniscus lenses with different specifications to test at the moment. Therefore, future studies are needed to validate the measurement results.

Our novel imaging technology holds tremendous promise in the detection and treatment assessment of ocular diseases, despite some limitations. One of the limitations is the need for a trained and skilled operator who can stably place the contact surface on the cornea to acquire images. Although the scanning pivot point is set up in advance to allow all the incident beams to pass through the iris plane and avoid the vignetting artifacts, long imaging depth is still required to prevent aliasing especially when imaging adults. The fast spectrum sweeping speed and the long imaging range required for our application has pushed the digitizer performance to the limit. Alternative OCT imaging technology such as circular ranging OCT might be needed to achieve shorter imaging time and longer imaging depth [57]. The optical performance in most of the scanning angles in the current system is already within or close to the diffraction limit. A larger beam size on the pupil will provide better transverse resolution that will theoretically be suitable for OCT angiography application, which requires a larger aperture galvanometer scanner and new optical design. However, a larger aperture galvanometer scanner has limited scanning speed, and alternative scanning pattern might be required [58], [59]. Moreover, the increase in numerical aperture may result in lower transverse resolution unless the optical design is optimized across all the scanning angles. Finally, a more accurate model eye with various axial eye lengths, as well as transverse resolution and FOV markers, would be very helpful in fully validating the system performance and calibrating the volumetric measurement.

## V. Conclusion

We demonstrated a contact handheld SS-OCT system with an unprecedented single shot of 140° FOV, extending the imaging area to the peripheral termination of the retina at the ora serrata. The detailed morphologies of the peripheral retina acquired by our novel imaging technology are valuable in many clinical applications, such as ROP, retinoblastoma. Coats disease, retinal detachment, etc. Alongside visualizing the pathologies in the peripheral retina, the handheld panretinal SS-OCT with contact approach may also be used to measure axial eye length, allowing for the quantitative assessment of disease progression and regression after treatment. Diseases such as retinoblastoma can be measured by tumor size.

As such, the visualization of the retinal periphery is increasingly used to diagnose pediatric ophthalmic diseases. The expanded FOV is conducive to identifying clinically inapparent pathologies in the peripheral retina. Clinicians particularly benefit from the peripheral OCT when assessing and diagnosing pathologies starting from the peripheral retina. Therefore, the introduction of the novel OCT imaging technology might herald an era in which the details in the peripheral retina will be depicted sufficiently and efficiently, opening a new window for understanding disease mechanisms.

## Figures and Tables

**TABLE I:** Optical parameters of Gullstrand eye model of new-born infants

Ocular segment	Spacing (mm)	Refractive index
CT	d_1_=0.75	1.377
ACD	d_2_=1.85	1.336
LT	d_3_=3.7	1.43
VCD	d_4_=11.0	1.334
RT^a^	d_5_=0.15	1.346

a: the parameter of retina from [34].

CT: Corneal Thickness. ACD: Anterior Chamber Depth. LT: Lens Thickness. VCD: Vitreous Chamber Depth. RT: Retinal Thickness.

**TABLE II:** Subjects’ diagnostic information

	Infant subjects
Total subjects	67
Diagnosis	ROP (45)
	Retinoblastoma (4)
	Retinal Detachment (5)
	Coats Disease (2)
	Incontinentia Pigmenti (5)
	Lattice Degeneration (1)
	Familial Exudative Vitreoretinopathy (1)
	Persistent Fetal Vasculature (1)
	Retinal Hemangioblastoma (1)

**TABLE III:** Tumor size of retinoblastoma at different positions

	Length (mm)	Width (mm)	Height(mm)
Tumor 1 (T1^a^)	6.37	3.05	1.41
Tumor 2 (T2)	5.2	4.04	1.08
Tumor 3 (T3)	5.48	4.71	1.56
Tumor 4 (T4)	9.08	7.14	2.84

a: The labels of tumor (T1-T4) are shown in Fig. 4(b).

**TABLE IV:** Axial length of the eye from premature infants

	GA	PMA	Axial length (mm)
Subject 1	23w4d^a^	37w5d	17.17
Subject 2	26w6d	53w2d	18.13
Subject 3	23w	34w6d	17.59
Subject 4	25w2d	35w5d	17.31
Subject 5	24w4d	33w1d	17.12
Subject 6	23w5d	33w3d	17.82
		34w3d	17.93
		35w3d	18.01
		37w5d	18.11
		40w3d	18.37
		41w3d	18.70

a: 23w4d refers to 23 weeks and four days. Others are similar.

GA: Gestational age. PMA: Postmenstrual age.
